# In Vitro and In Vivo Genotoxicity Assessments and Phytochemical Analysis of the Traditional Herbal Prescription Siryung-Tang

**DOI:** 10.3390/molecules27134066

**Published:** 2022-06-24

**Authors:** Chang-Seob Seo, Mi-Sook Jung, Hyeun-Kyoo Shin, Mee-Young Lee

**Affiliations:** 1 KM Science Research Division, Korea Institute of Oriental Medicine, Daejeon 34054, Korea; csseo0914@kiom.re.kr (C.-S.S.); hkshin@kiom.re.kr (H.-K.S.); 2 Advanced Toxicity Evaluation Team 2, Biotoxtech Co., Ltd., Cheongju 28115, Korea; mszheng@biotoxtech.com; 3 KM Convergence Research Division, Korea Institute of Oriental Medicine, Daejeon 34054, Korea

**Keywords:** genotoxicity, phytochemical analysis, siryung-tang, chailing-tang, sairei-to

## Abstract

Siryung-tang (SRT) is a traditional herbal prescription containing Oryeong-san and Soshiho-tang that is used to treat digestive system diseases. We performed safety evaluations of SRT based on genotoxicity and developed an assay for quality control using high-performance liquid chromatography with a photodiode array detector. Genotoxicity was evaluated based on bacterial reverse mutation (*Salmonella typhimurium* TA1535, TA98, TA100, and TA1537, and *Escherichia coli* WP2 uvrA), chromosomal aberration (Chinese hamster lung cells), and micronucleus (mouse) tests. Quality control analysis was conducted using a SunFire C_18_ column and gradient elution with a distilled water–acetonitrile mobile phase system containing 0.1% (*v*/*v*) formic acid for 12 markers (5-(hydroxy-methyl)furfural, 3,4-dihydroxybenzaldehyde, liquiritin apioside, liquiritin, coumarin, baicalin, wogonoside, cinnamaldehyde, baicalein, glycyrrhizin, wogonin, and atractylenolide III). SRT showed no genotoxicity in three tests. Ames tests showed that SRT at 313–5000 μg/plate did not significantly increase the number of revertant colonies with or without metabolic activation among five bacterial strains. Moreover, in vivo micronucleus testing showed that SRT did not increase the frequency of bone marrow micronuclei. The number of chromosomal aberrations associated with SRT was similar to that observed in the negative controls. The 12 markers were detected at 0.04–16.86 mg/g in a freeze-dried SRT sample and completely eluted within 45 min. The extraction recovery was 95.39–104.319% and the relative standard deviation value of the precision was ≤2.09%. Our study will be used as basic data for the safety and standardization of SRT.

## 1. Introduction

Herbal prescriptions containing numerous components are used for treating or preventing various diseases [1]. The demand for and interest in traditional medicines are increasing because they contain two or more herbal medicines extracted using water and have few side effects [2]. However, it is necessary to consider the appropriate dosage, administration method, and administration period, as well as the patient’s condition and constitution, in order to prescribe and take herbal prescriptions in a safe manner.

Siryung-tang (SRT; also known as Chailing-tang in Chinese and Sairei-to in Japanese), is a traditional herbal prescription composed of two different traditional Korean medicines (Oryeong-san and Soshiho-tang) that is used for the treatment of fever and diarrhea [3]. SRT was first recorded in Se-ui-deuk-hyo-bang, which is a medical book written based on the treatment experience of Wilin Wei during the Yuan Dynasty, and in Donguibogam, which is the most famous Korean medical book, compiled by Jun Heo during the Joseon Dynasty [3,4]. In Japan, SRT is an experiential prescription formula (Honchokeikenho) used by patients with certain health conditions or symptoms of gastritis, indigestion, edema, food poisoning, ulcerative colitis, recurrent miscarriages, and autoimmune diseases [5]. According to these documents, SRT is composed of the following 11 herbal medicines: *Bupleurum falcatum* L., *Alisma orientale* Juzep., *Atractylodes japonica* Koidz., *Polyporus umbellatus* Fries, *Poria cocos* Wolf, *Pinellia ternata* Breit., *Scutellaria baicalensis* Georgi, *Panax ginseng* C.A. Mey., *Glycyrrhiza uralensis* Fisch., *Cinnamomum cassia* (L.) J. Presl, and *Zingiber officinale* Roscoe.

The pharmacological activity of SRT has been reported in the treatment of ulcerative colitis and diabetic nephropathy, in addition to its hepatoprotective effects [6,7,8,9,10]. Ohno et al. [11] reported the renal protective effect of SRT on gentamicin-induced nephrotoxicity, and Katami et al. [12] investigated the genotoxicity of 128 types of Kampo medicine, including SRT. Details of the biological activities of the traditional herbal medicine SRT have been reported, but insufficient scientific evidence is available to assess its safety. Furthermore, assessments of SRT standardization for consistent efficacy and quality control studies have not been fully reported.

Therefore, in the present study, we conducted genotoxicity tests based on bacterial reverse mutation (Ames tests), in vitro chromosomal aberration tests, and in vivo micronucleus tests according to the guidelines established by the Organization for Economic Co-operation and Development (OECD) in ENV/MC/CHEM(98)17 (as revised in 1997) for the testing of chemicals in accordance with the current Good Laboratory Practice (GLP) regulations (KFDA 2018-93) [13]. In addition, a simultaneous analysis method was developed using high-performance liquid chromatography (HPLC) coupled with a photodiode array (PDA) detector for SRT quality evaluation.

## 2. Results

### 2.1. Genotoxicity Evaluation for SRT

#### 2.1.1. Ames Test

According to the dose determination test results, the doses for strains TA98, TA100, TA1535, TA1537, and WP2 uvrA (pKM101) were 5000, 2500, 1250, 625, and 313 μg/plate, respectively.

**Figure 1:**
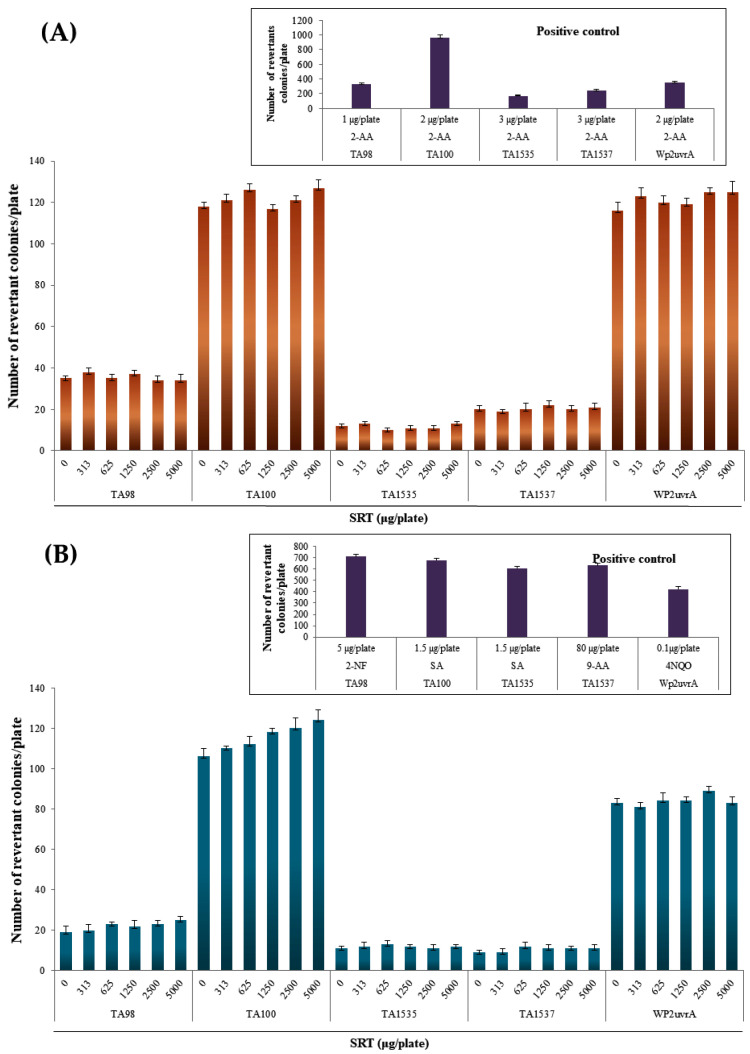
Effects of the SRT extract in the Ames test (**A**) with (+S9 mix) and (**B**) without (–S9 mix) metabolic activation. Positive controls: SA, sodium azide; 2-NF, 2-nitrofluorene; 9-AA, 9-aminoacridine; 4NQO, 4-nitroquinoline N-oxide; 2-AA, 2-aminoanthracene; BP, benzopyrene.

In the SRT group, the number of reverted colonies at all doses for each strain did not exceed twice that in the negative control group, regardless of the presence or absence of metabolic activation (Figure 1). The number of reverted colonies in the positive control group for each strain was more than two times higher than that in the negative control group. Growth inhibition and precipitation by SRT were not observed at all doses for each strain in the absence and presence of metabolic activation (Figure 1). The reproducibility of the gene mutagenesis results was confirmed, and the lack of growth inhibition was demonstrated at four or more dose levels.

The average numbers of reverted colonies in the negative control group and positive control group were within the ranges of historical control data, and the number of reverted colonies in the positive control group for each strain was more than two times higher than that in the negative control group. In addition, contamination by various bacteria was not detected, thereby indicating that the test was performed appropriately.

#### 2.1.2. Chromosome Aberration Test Results

Doses of 625, 313, 156, and 78.1 μg/mL SRT were administered. Relative population doubling (RPD) was greater than 85.6 at doses of 0, 78.1, 156, 313, and 625 μg/mL in the absence of metabolic activation. In addition, in the presence of metabolic activation, RPD was more than 71.0 at doses of 0, 78.1, 156, 313, and 625 μg/mL (Table 1). No statistically significant differences in the frequency of occurrence of cells with chromosomal abnormalities were found in the absence and presence of metabolic activation compared with the negative control group. In the positive control group for each treatment, a statistically significant increase was confirmed in the frequency of occurrence of cells with structural abnormalities compared with the negative control group (*p* < 0.01) (Table 1). In the negative control group, the frequency of occurrence of cells with chromosomal structural aberrations was within the control range for historical control data (data not shown). The frequency of occurrence of cells with chromosomal structural abnormalities in the positive control group was within the control range for historical control data, and a statistically significant increase was confirmed compared with the negative control group.

#### 2.1.3. Micronucleus Test

During the test period, abnormalities in general symptoms caused by SRT were assessed at all doses in the SRT group. No statistically significant changes in body weight were observed at all doses in the SRT group and positive control group compared with the negative control group. The PCE/(PCE + NCE) ratio is used as an indicator of cytotoxicity. No statistically significant difference in the ratio of MNPCE/PCE was found compared with the negative control group. In the SRT group, no statistically significant difference in the ratio of MNPCE/PCE was found compared with the negative control group at all doses (Table 2). MNPCE/PCE means the ratio of micronuclei observed in PCE, and if there is a significant difference, micronuclei are induced in the bone marrow. In the positive control group, a statistically significant increase in the frequency of MNPCE/PCE ratio was found compared with the negative control group (*p* < 0.01). No statistically significant difference in the ratio of polychromatic red blood cells relative to total red blood cells was found compared with the negative control group.

### 2.2. Quality Assessment of SRT by HPLC–PDA

#### 2.2.1. Optimization of HPLC–PDA Conditions for Quality Control Analysis of SRT

**Figure 2:**
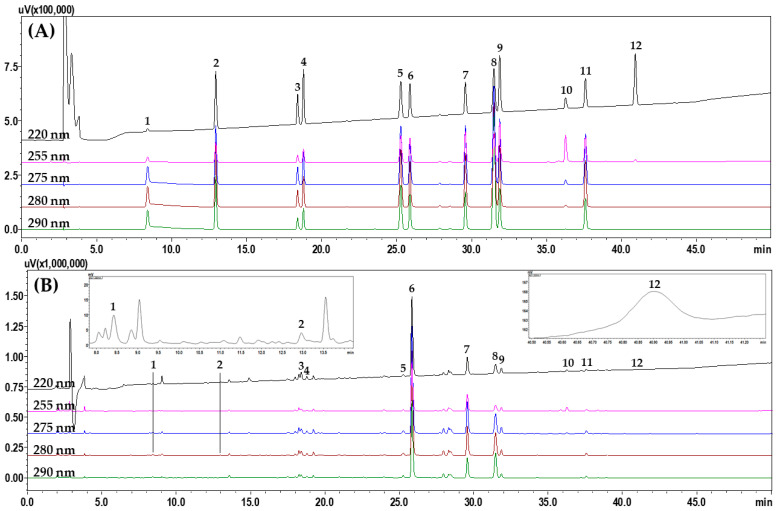
Representative HPLC chromatograms obtained for (**A**) standard solution and (**B**) 70% methanol extract of SRT water extract. The indicated peaks are 5-(hydroxy-methyl)furfural (**1**), 3,4-dihydroxybenzaldehyde (**2**), liquiritin apioside (**3**), liquiritin (**4**), coumarin (**5**), baicalin (**6**), wogonoside (**7**), cinnamaldehyde (**8**), baicalein (**9**), glycyrrhizin (**10**), wogonin (**11**), and atractylenolide III (**12**).

To select suitable marker analytes for SRT quality control, HPLC analysis was performed after investigating 11 raw herbal medicines and their main components, as follows: saikosaponin A from *B. falcatum*; alisol B and alisol B acetate from *A. orientale*; atractylenolide I, II, and III from *A. japonica*; 5-(hydroxy-methyl)furfural, 3,4-dihydroxybenzaldehyde, 4-hydroxybenzaldehyde, ferulic acid, emodin, chrysophanol, and physcion from *P. umbellatus*; pachymic acid and polyporenic acid C from *P. cocos*; homogentisic acid and 3,4-dihydrobenzaldehyde from *P. ternata*; baicalin, wogonoside, baicalein, and wogonin from *S. baicalensis*; ginsenosides Rg1 and Rb1 from *P. ginseng*; liquiritin apioside, liquiritin, liquiritigenin, and glycyrrhizin from *G. uralensis*; coumarin, cinnamic acid, and cinnamaldehyde from *C. cassia*; and 6-gingerol from *Z. officinale* (Figure S1 in Supplementary materials). After selecting 12 of these components as the final marker analytes (5-(hydroxy-methyl)furfural, 3,4-dihydroxybenzaldehyde, liquiritin apioside, liquirin, coumarin, baicalin, wogonoside, cinnamaldehyde, baicalein, glycyrrhizin, wogonin, and atractylenolide III), we compared the separation patterns of the markers obtained with different columns (4.6 mm × 250 mm, 5 μm) from SunFire (Waters, Milford, MA, USA), Gemini (Phenomenex, Torrance, CA, USA), Capcell Pak UG80 (Shiseido, Tokyo, Japan), Quasar SPP (PerkinElmer, Shelton, CT, USA), and INNO (YoungJinBioChrom Co., Ltd., Seongnam, Korea), as well as different column temperatures (30 °C, 35 °C, 40 °C, and 45 °C) and acids (formic acid, trifluoroacetic acid, phosphoric acid, and acetic acid). The optimal separation conditions were determined as a water–acetonitrile mobile phase containing formic acid and a SunFire column at 40 °C. HPLC analysis using the optimized assay conditions eluted the 12 marker components, comprising 5-(hydroxy-methyl)furfural (**1**), 3,4-dihydroxybenzaldehde (**2**), liquiritin apioside (**3**), liquiritin (**4**), coumarin (**5**), baicalin (**6**), wogonoside (**7**), cinnamaldehyde (**8**), baicalein (**9**), glycyrrhizin (**10**), wogonin (**11**), and atractylenolide III (**12**) at 8.39, 12.93, 18.38, 18.77, 25.26, 25.87, 29.57, 31.4, 31.86, 36.25, 37.58, and 40.90 min, respectively, within 45 min (Figure 2).

#### 2.2.2. Validation of HPLC Method Developed for Quality SRT Control

As shown in Table S1, the retention factor (*k’*), relative retention (*α*), resolution (*Rs*), number of theoretical plates (*N*), and tailing factor (*Tf*) values of ≥2.00, ≥1.01, ≥55,709, ≥1.57, and 1.01–1.24, respectively, confirmed the suitability of the system for SRT analysis in this study. The calibration curve for each marker was prepared by measuring three times at seven different concentrations. The calibration curves for all of the markers were obtained as the regression equation (*y* = a*x* + b) based on the peak area (*y*) versus the corresponding concentration (*x*, μg/mL) for the injected standard solutions. As shown in Table 3, the regression analysis results, coefficient of determination (*r*^2^), limit of detection (LOD), and limit of quantification (LOQ) were satisfactory for all of the marker analytes. In the detection range, *r*^2^ was greater than 0.9998, with good and acceptable linearity. LOD and LOQ were calculated as 0.01–0.16 μg/mL and 0.03–0.50 μg/mL for all markers, respectively. Table 4 and Table 5 show the recovery test results and precision and repeatability test results, respectively. The average recoveries of the marker analytes were 95.39–104.31% and the relative standard deviation (RSD) value did not exceed 2.57%. According to the precision tests, the intra-day variation for the marker components had an RSD of ≤0.75% and inter-day variation an RSD of ≤2.09%, with accuracy range of 96.54–102.82% and 95.15–102.99%, respectively. The RSD values for the retention time and peak area were ≤0.07% and ≤0.90%, respectively (Tables S2 and S3). These results suggest that the HPLC method developed in this study is appropriate and reliable for SRT quality assessment.

#### 2.2.3. Quantification of the 12 Marker Analytes in SRT Samples

The validated HPLC–PDA assay was successfully applied for SRT quality control analysis and used to quantify 12 marker components in SRT samples. In the 70% methanol extracts from the SRT water decoctions, 12 markers were detected at 0.04–16.86 mg/g (Table 6). Among the markers, the baicalin (major compound of *S. baicalensis*) content was highest at 16.63–16.86 mg/g. Our results show that this is similar to the results of previous studies reporting the qualitative analysis of SRT [14,15].

## 3. Discussion

Most herbal products are considered safe if used at the recommended doses, but undesirable effects can still occur [16]. Genetic toxicology analysis is an important component of nonclinical safety evaluations for drugs and an important link for moving drug candidates from the discovery to the clinical stage [17]. In the present study, the results obtained based on three types of genetic toxicity test, i.e., the bacterial reverse mutation test, the in vitro mammalian chromosome aberration test, and the mammalian erythrocyte micronucleus test, were negative for SRT. To identify an appropriate toxicity range, preliminary range-finding assays were conducted over a broad range of concentrations. The potential mutagenicity of SRT was determined by the Ames test, which detected no significant increase in the number of revertant colonies after exposure to different SRT concentrations. Based on these results, this means that SRT does not contain complexes that significantly induce mutagenicity at up to 5000 µg/plate. However, since the Ames test result alone cannot be said to indicate a non-mutagenic substance, in vivo experiments such as comet assays are required. The chromosomal aberration test is used to identify materials that induce structural chromosomal aberrations in cultured mammalian cells. In the present study, the chromosomal aberration assay was conducted in the presence or absence of S9 metabolic activation in Chinese hamster lung (CHL) cells. Chromosomal mutations are the cause of many human genetic diseases [18], and strong evidence indicates that chromosomal mutations and related mechanisms that cause alterations in tumor suppressor genes and oncogenes in somatic cells are concomitant in cancer development in humans. Based on our results, we conclude that SRT is non-mutagenic. Furthermore, mice treated with various doses of SRT did not exhibit weight loss or increases in abnormal micronuclei. The formation of micronuclei is an indication of induced chromosome damage [19], and an elevated frequency of micronucleated polychromatic erythrocytes denotes chromosomal damage [20]. Mutagenicity testing using the Ames test, chromosomal aberration test, and micronucleus test (the three-battery test) has high value for screening carcinogenicity in rodents when positive results are obtained [21]. Our results demonstrate that SRT may be suitable for further development as a drug candidate with various potential applications.

## 4. Materials and Methods

### 4.1. Plant Materials

The 11 medicinal herbs used for preparing SRT are shown in Table S4. The plant species were confirmed using the “The Plant List” website (http://www.theplantlist.org/, accessed on 13 May 2022). All of the raw materials (2020–KE89–1 to 2020–KE89–11) were purchased from Kwangmyungdag Medicinal Herbs (Ulsan, Korea) in August 2020 and used after morphological examination by Dr. Goya Choi, Korea Institute of Oriental Medicine (Naju, Korea) according to the guidelines of “The Dispensatory on the Visual and Organoleptic Examination of Herbal Medicine” [22].

### 4.2. SRT Water Extract Preparation

SRT water extract for use in this study was prepared according to the preparation protocol of the previously reported study [23]. In other words, 11 herbal medicines were mixed at the weights shown in Table S1 (4.0 kg), before adding 40 L of distilled water and extracting with an electric extractor (COSMOS-660; Kyungseo E&P, Incheon, Korea) for 2 h at 100 °C. The extract was filtered through a sieve (mesh size: 53 μm) and then dried using a freeze-dryer (PVTFD100R; IlShinBioBase, Dongducheon, Korea) to obtain a powdered sample. As a result, a sample weighing 685.0 g (yield: 17.1%) was obtained. The prepared sample was kept refrigerated (4 °C) and used for HPLC analysis and genotoxicity studies.

### 4.3. Genotoxicity Evaluation for SRT

#### 4.3.1. Ames Test

The Ames test was performed to determine the genetic mutagenicity of SRT using *Salmonella typhimurium* bacterial strains and a tryptophan-requiring *Escherichia coli* bacterial strain in accordance with the GLP regulations for ‘Non-clinical Trial Management Standards’ (Ministry of Food and Drug Safety Notice No. 2018-93) and OECD guidelines for the Testing of Chemicals, 471, Bacterial Reverse Mutation Test (OECD; 26 June 2020) [24]. Testing was performed using pre-culture methods. After confirming the strain characteristics, each frozen strain was thawed at room temperature, inoculated in nutrient broth medium, and cultured with shaking (37 °C, 130 rpm; BS-31 Jeio Tech Co., Ltd., Daejeon, Korea). After pre-culture, the absorbance of each strain was measured using an ultraviolet/visible spectrophotometer (measurement wavelength: 660 nm; V-550; Jasco, Tokyo, Japan) and employed for testing after confirming that the number of bacteria exceeded 1 × 10^9^ cells/mL. As positive controls, 2-nitrofluorene (2-NF), 2-aminoanthracene, 9-aminoacridine, 4-nitroquinoline N-oxide, and sodium azide were used to determine the SRT test dose, where 5000 μg/plate was employed as the highest dose, as recommended in the guidelines, and azeotrope 4 was applied for the following doses, with test substance groups of 1250, 313, 78.1, 19.5, and 4.88 μg/plate. In the absence of metabolic activation, 100 μL of SRT, the negative control, or positive control was placed into a tube before adding 500 μL of 0.1 mol/L phosphate buffer (pH 7.4) and 100 μL of each strain suspension and incubating with shaking for 20 min at 37 °C. After shaking, the top agar was added for *Salmonella* strains TA98, TA100, TA1535, and TA1537, and 2 mL of the top agar for *E. coli* WP2 uvrA (pKM101) strain, before vortexing, layering on a minimum glucose agar plate medium, and allowing to set at room temperature. In the presence of metabolic activation, 500 μL of S9 mix was added instead of 500 μL of 0.1 mol/L phosphate buffer (pH 7.4). To confirm the presence or absence of contamination by various bacteria, 500 μL of 0.1 mol/L phosphate buffer (pH 7.4) and 500 μL of S9 mix were placed in each tube. The presence or absence of colony formation due to microbial contamination was confirmed. After treating with the top agar, the plate was inverted and cultured for 48 h in an incubator at 37 °C (DK-LI020-P; Daiki Scientific Co., Ltd., Seoul, Korea). The precipitation of SRT was visually observed and recorded after SRT treatment, and the number of reverted colonies was counted. After culturing, the number of reverted colonies was automatically measured with an automatic colony counter instrument (ProtoCOL3; Synbiosis, Cambridge, UK) or visually counted. Visual counting was performed if we considered that the automatic measurement was not accurate.

#### 4.3.2. Chromosome Aberration Test

The chromosome aberration assay was conducted with CHL cells (American Type Culture Collection, Manassas, VA, USA), which were maintained in Eagle’s minimum essential medium (Lonza, Walkersville, MD, USA) containing 10% fetal bovine serum (Invitrogen, Carlsbad, CA, USA). Cells stored in frozen liquid nitrogen were thawed and the cell morphology was observed when the cells covered more than 70–80% of the bottom of the culture flask. The cells were separated from the bottom of the flask by treating with 0.25% trypsin ethylenediaminetetra acetic acid (EDTA) solution. The cell suspension was placed in a 50 mL tube and centrifuged at 1000 rpm for 5 min. Cells passaged twice or more were used, and the cultured cells were prepared as a cell suspension with 5 × 10^4^ cells/mL. The dose determination test was conducted by dispensing 2 mL of the cell suspension into a six-well plate (2 mL/well; Nunc, Roskilde, Denmark), and the main test was conducted on 60 mm plates (5 plate, BD Biosciences, San Diego, CA, USA) and six-well plates, before incubating for 1 day at 37 °C with 5% CO_2_. The test concentrations of SRT were 5000, 2500, 1250, 625, 313, 156, 78.1, 39.1, and 19.5 μg/mL. Positive controls comprising mitomycin C (MMC) and benzo[a]pyrene were stored at −80 °C to −60 °C (OPR-DFU-657CEV, Operon, Gimpo, Korea). For the cytotoxicity tests, a satellite control group was prepared with one well for each test. SRT precipitation was observed for each dose at the time of treatment, the end of treatment, and the end of culture. After SRT treatment, the pH and osmolality of the negative control group and the highest SRT dose were measured.

According to the results, the pH and osmotic pressure with the highest SRT dose did not change more than 1.0 and 50 mOsm/kg, respectively, compared with the negative control group. No difference in the color of the medium due to a change in pH was observed. The satellite control group was treated with SRT and the SRT group was counted using a hemocytometer to calculate the RPD after culture was terminated. RPD was calculated as follows:

Population doubling = [log (post-treatment cell number/initial cell number)]/log2(1)

(2) $$\;\mathrm{RPD}\;\left(\%\right)=\frac{\mathrm{No}.\;\mathrm{of}\;\mathrm{Population}\;\mathrm{doubling}\;\mathrm{in}\;\mathrm{treated}\;\mathrm{cultures}}{\mathrm{No}.\;\mathrm{of}\;\mathrm{Population}\;\mathrm{doubling}\;\mathrm{in}\;\mathrm{control}\;\mathrm{cultures}}\;\times 100\;\left(\%\right)$$

To prepare each sample, colcemid solution (Gibco, Grand Island, NY, USA) was added to obtain a final concentration of 0.2 μg/mL 2 h before the end of culture to stop cell division in the metaphase. After the completion of culture, cells were removed from the bottom of the plate by means of treatment with 0.25% trypsin EDTA solution (Gibco) and centrifuged at 1000 rpm for 5 min (FLETA 5; Hanil Science Industrial Co., Ltd., Incheon, Korea). After removing 5 mL of the aqueous solution, the supernatant was mixed with 0.075 mol/L KCl and incubated at 37 °C for 20 min. After adding 1 mL of cooling fixative (methanol: acetic acid = 3:1), centrifugation was performed at 1000 rpm for 5 min and the supernatant was removed. The fixing operation was repeated once more. After suspending the cells in a small amount of cooling fixative, one drop was dropped on each of the two glass slides to make one sample slide. After drying, a code number was written on the glass slide. Next, 3% Giemsa dye was applied for 20 min, before washing with ultrapure water, drying, and sealing with an encapsulant (Entellan^®^ New; Merck, Darmstadt, Germany).

Specimens were observed on the prepared slides. At the target dose for chromosome observation, three doses were tested, and more than 300 metaphase phases were observed per dose for each treatment. The 300 fragmented metaphase phases per dose were observed under a microscope at 600× magnification (BX51; Olympus, Tokyo, Japan). Chromosomal abnormalities were classified as structural abnormalities, numerical abnormalities, and others. The observed structural abnormalities comprised chromatid break, chromatid exchange, chromosome break, chromosome exchange, chromatid gap, chromosome gap, and fragmentation. In one metaphase phase, multiple gaps and cleavages were recorded as fragmentation.

A gap was defined as a non-staining region narrower than the chromatid width. In addition, polyploidy and nuclear internalization were recorded. Cells with one or more abnormalities were counted as abnormal cells and each type was recorded. Abnormal cells with or without gaps were recorded separately.

In other cases, the type and number were recorded, unless they were included in the structural abnormality and numerical data. This test was conducted according to OECD Guideline No. 473 In Vitro Mammalian Chromosome Aberration Test (Adopted: 29 July 2016) and OECD Principles of GLP (as revised in 1997) [25].

#### 4.3.3. Micronucleus Test

The micronucleus test was conducted according to OECD Guideline No. 474 Mammalian Erythrocyte Micronucleus Test (26 September 2014) and GLP regulations (2018-93) [26]. Seven-week-old specific pathogen-free male and female ICR mice (dose finding test: male *n* = 17, female *n* = 17/main test male *n* = 38) were purchased from Orient Bio Company (Seongnam, Korea). According to the dose finding test, no abnormalities, general symptoms, or death were observed due to SRT at all doses in both sexes. Therefore, the main test was conducted with males known to be susceptible to micronucleus induction. The SRT test doses were 5000, 2500, and 1250 mg/kg. The dose of SRT was used at 5000, 2500 and 1250 mg/kg. MMC was used as the positive control at 2 mg/kg/day and was dissolved in sterile distilled water before use. ICR mice were orally administered once each day with SRT at doses of 5000, 2500, and 1250 mg/kg. MMC was administered at a dose of 2 mg/kg by intraperitoneal injection as a positive control.

During the administration period, general symptoms comprising appearance, behavior, extraction, and death were observed two times each day (immediately before administration and immediately after administration), and once each day during other periods.

The animals were subjected to euthanasia by cervical dislocation after administration of the test substance to prepare specimens. After removing the femur and attached muscle, both ends were cut with scissors and perfused with 200 μL of fetal bovine serum (Lot No.: 2190536P; Gibco) to collect bone marrow cells. The bone marrow cell suspension was centrifuged for 5 min at 1000 rpm and 4 °C (Micro17TR; Hanil Science Industrial Co., Ltd., Gimpo, Korea). The supernatant was removed and the precipitated bone marrow cells were suspended, before placing a small amount on a glass slide. After drying the glass slide, it was fixed with methanol and stained with 3% Giemsa staining solution (prepared with 0.01 mol/L Sörenson phosphate buffer (pH 6.8)) for 30 min. The stained slides were washed with 0.01 mol/L Sörenson phosphate buffer (pH 6.8) and 0.004% citric acid aqueous solution, before drying and sealing with a sealing agent (Entellan^®^ New; Merck).

The specimen slides were observed under an optical microscope at 600× magnification (BX51, CH30; Olympus). In total, 4000 polychromatic erythrocytes were observed per slide, and the rate of appearance of micronucleated polychromatic erythrocytes was calculated for each individual. In addition, we checked whether the ratio of polychromatic red blood cells relative to the total number of red blood cells in the SRT group was ≥20% that in the negative control group. Next, 500 red blood cells per individual were observed and the ratio of polychromatic red blood cells relative to the total red blood cells was calculated as an index of myeloid cell proliferation inhibition.

### 4.4. HPLC Analysis for Quality Control of SRT

#### 4.4.1. Chemicals and Reagents

Compounds **1**–**12** (Figure S2) for quality control of SRT were purchased from natural product manufacturing companies: compounds **1** (Cat No. W501808, purity ≥ 99.0%), **2** (Cat No. D108405, purity 97.0%), and **5** (Cat No. C4261, purity ≥ 99.0%) from Merck KGaA (Darmstadt, Germany); compounds **3** (Cat No. DR10690, purity ≥ 98.0%), **6** (Cat No. DR10626, purity 98.5%), **7** (Cat No. DR10630, purity 98.9%), **9** (Cat No. DR10625, purity 99.4%), and **12** (Cat No. DR11040, purity ≥ 98.0%) from Shanghai Sunny Biotech Co., Ltd. (Shanghai, China); compound **4** (Cat No. BP0874, purity 99.6%) from Biopurify Phytochemicals Ltd. (Chengdu, China); and compounds **8** (Cat No. 031-03456, purity 98.0%), **10** (Cat No. 070-05161, purity 99.4%), and **11** (Cat No. 236-02321, purity 98.9%) from Fujifilm Wako Pure Chemical Co. (Osaka, Japan).

HPLC-grade methanol (Cat No. 9093-88), acetonitrile (Cat No. 9017-88), distilled water (Cat No. 4218-88), and formic acid (Cat No. 063-04192, purity 99.5%) for HPLC were purchased from J.T.Baker (Phillipsburg, NJ, USA) or Fujifilm Wako Pure Chemical Co. (Osaka, Japan).

#### 4.4.2. Simultaneous Analysis of the 12 Marker Components Using HPLC

Simultaneous analysis for quality control using the 12 markers in the SRT sample was performed by a Prominence LC-20A series (Shimadzu, Kyoto, Japan), which was combined with a PDA detector capable of scanning 190–800 nm simultaneously and LabSolution software (version 5.53, SP3, Shimadzu, Kyoto, Japan) for system control, as shown in Table S5.

The test solution and the standard solution for HPLC analysis of the SRT sample were prepared at 10.0 mg/mL and 1.0 mg/mL using 70% methanol and methanol, respectively. All of these solutions were filtered with a 0.2 μm membrane filter (Pall Life Sciences, Ann Arbor, MI, USA) before analysis.

#### 4.4.3. System Suitability Test and Method Validation of the Developed HPLC Analytical Method

A system suitability test was carried out to establish the suitability of the system used in the analytical test in the analysis, and it was confirmed through the *k’*, *α*, *Rs*, *N*, and *Tf*.

Method validation of the developed HPLC analytical method was evaluated through the linearity, LOD, LOQ, accuracy, and precision. LOD and LOQ were calculated using the following equations: LOD = 3.3 × *σ*/*S* and LOQ = 10 × *σ*/*S*, where *σ* and *S* are the standard deviation of the *y*-intercept and the slope of the calibration curve, respectively. Accuracy was evaluated by the recovery test, and this test was conducted by adding a standard solution of three different concentrations (low, medium, and high) to a known SRT sample, and calculating the extraction recovery from the following equation: Recovery (%) = measured concentration/spiked concentration × 100%. Precision was evaluated through repeatability and intra-day and inter-day precisions. Repeatability was confirmed by the RSD (RSD (%) = standard deviation (SD)/mean × 100%) of retention time and peak area after 6 repeated measurements using a standard solution mixed with 12 marker compounds. The intra-day precision and the inter-day precision were evaluated by RSD after measuring for one day and three consecutive days for three different concentrations using a standard solution, respectively.

### 4.5. Statistical Analysis

The frequency of appearance of cells with chromosomal aberrations was statistically analyzed using SAS (version 9.3, SAS Institute Inc., Cary, NC, USA). Fisher’s exact test was performed on the frequency of appearance of cells with chromosomal abnormalities (gap not included) to test the significance between the negative control group and the test substance group and between the negative control group and the positive control group.

Statistical analysis was performed using SAS (version 9.3, SAS Institute Inc., Cary, NC, USA) for the frequency of micronuclear polychromatic red blood cells, the ratio of polychromatic red blood cells to total red blood cells, and changes in body weight. The Kruskal–Wallis test and Mann–Whitney U test were performed for the frequency of micronuclear polychromatic red blood cells to test the significant difference between the negative control group and the test substance group and the significant difference between the negative control group and the positive control group. The dose dependence of the test substance group was tested for the significance between the test substance groups by conducting the Cochran–Armitage trend test. The Bartlett test was performed on the frequency and body weight of polychromatic erythrocytes for total red blood cells, and the equality of variance between the negative control group and the test substance group was tested 24 h after administration. Equal variance was recognized, and significance was confirmed by performing one-way analysis of variance (ANOVA). For comparison between the negative control group and the positive control group 24 h after administration, and the negative control group and the test substance group 48 h after administration, the Folded-F test was performed to test for equality of variance. Equal variance was recognized, and the significance was confirmed by performing Student’s *t*-test.

## 5. Conclusions

This study was conducted to establish basic data for safety evaluation and quality control of SRT, a traditional prescription mainly used for the treatment of ulcerative colitis. Genotoxicity was verified through the Ames test using the *S. typhimurium* strains TA1535, TA98, TA100, and TA1537, and the *E. coli* strain WP2 uvrA, the chromosomal aberration test using CHL cells, and the micronucleus test using mouse cells. The results of the toxicology studies showed no indications for safety concerns in three genetic toxicity studies. In addition, an analysis method for quality control was developed, and this method was verified through linearity, LOD, LOQ, recovery, and precision. These results will be used as basic data for the safety and quality control of traditional herbal prescriptions.

## Figures and Tables

**Table 1:** Chromosomal aberrations due to SRT in Chinese hamster lung cells.

Group	Conc. (μg/mL)	RPD (%)	S9 Mix	Treatment Time (h)	No. of Cells Analyzed	Number of Cells with Structural Aberrations									Number of Cells with Numerical Aberrations		
						ctb	csb	cte	cse	frg	gap		Total (%)		End	Pol	Total (%)
											ctg	csg	gap–	gap+			
SRT	0	100	–	6–18	150	0	0	0	0	0	0	0	0 (0.0)	0 (0.0)	0	0	0 (0.0)
						0	0	0	0	0	0	0			0	0	
	78.1	92.8	–	6–18	150	Not observed											
	156	90.1	–	6–18	150	0	0	0	0	0	0	0	0 (0.0)	1 (0.3)	0	0	0 (0.0)
						0	0	0	0	0	1	0			0	0	
	313	87.3	–	6–18	150	0	0	0	0	0	0	0	1 (0.3)	1 (0.3)	0	0	0 (0.0)
						0	0	1	0	0	0	0			0	0	
	625	85.6	–	6–18	150	0	0	0	0	0	0	0	0 (0.0)	0 (0.0)	0	0	0 (0.0)
						0	0	0	0	0	0	0			0	0	
MMC	0.1	58.5	–	6–18	150	9	0	26	0	0	0	0	56 ** (18.7)	58 (19.3)	0	0	0 (0.0)
						8	0	23	0	0	2	0			0	0	
SRT	0	100	+	6–18	150	0	0	0	0	0	0	0	0 (0.0)	0 (0.0)	0	0	0 (0.0)
						0	0	0	0	0	0	0			0	0	
	78.1	93.8	+	6–18	150	Not observed											
	156	88.9	+	6–18	150	0	0	0	0	0	0	0	1 (0.3)	1 (0.3)	0	0	0 (0.0)
						0	0	1	0	0	0	0			0	0	
	313	85.5	+	6–18	150	0	0	0	0	0	0	0	0 (0.0)	1 (0.3)	0	0	0 (0.0)
						0	0	0	0	0	1	0			0	0	
	625	84.3	+	6–18	150	0	0	0	0	0	0	0	0 (0.0)	0 (0.0)	0	0	0 (0.0)
						0	0	0	0	0	0	0			0	0	
B[a]P	20	50.7	+	6–18	150	5	0	25	0	0	0	0	58 ** (19.3)	58 (19.3)	0	0	0 (0.0)
						10	0	24	0	0	0	0			0	0	
SRT	0	100	–	24–0	150	0	0	0	0	0	0	0	0 (0.0)	0 (0.0)	0	0	0 (0.0)
						0	0	0	0	0	0	0			0	0	
	78.1	89.7	–	24–0	150	Not observed											
	156	88.0	–	24–0	150	0	0	0	0	0	0	0	1 (0.3)	1 (0.3)	0	0	0 (0.0)
						1	0	0	0	0	0	0			0	0	
	313	82.3	–	24–0	150	0	0	0	0	0	0	0	0 (0.0)	0 (0.0)	0	0	0 (0.0)
						0	0	0	0	0	0	0			0	0	
	625	71.0	–	24–0	150	0	0	0	0	0	0	0	0 (0.0)	0 (0.0)	0	0	0 (0.0)
						0	0	0	0	0	0	0			0	0	
MMC	0.1	52.7	–	24–0	150	9	0	32	0	0	0	0	70 ** (23.3)	71 (23.7)	0	0	0 (0.0)
						13	0	27	0	0	1	0			0	0	

ctg: chromatid gap, csg: chromosome gap, ctb: chromatid break, cte: chromatid exchange, csb: chromosome break, cse: chromosome exchange, frg: fragmentation, end: endoreduplication, pol: polyploidy, MMC: mitomycin C, B[a]P: benzo[a]pyrene, RPD: relative population doubling, Trt-Rec time: treatment-recovery time, gap–: total number of cells with structural aberrations excluding gaps, gap+: total number of cells with structural aberrations including gaps. ** *p* < 0.01: significant difference compared with negative control according to Fisher’s exact test.

**Table 2:** Results of micronucleus assays in male ICR mice.

Group	Dose (mg/kg)	Hours after Dosing	PCE/(PCE + NCE) % (Mean ± SD)	MNPCE/PCE % (Mean ± SD)
Negative control	0	24	33.4 ± 0.59	0.025 ± 0.025
		48	33.2 ± 0.63	0.030 ± 0.021
SRT	1250	24	32.4 ± 1.57	0.030 ± 0.033
	2500	24	32.5 ± 1.61	0.025 ± 0.000
	5000	24	32.7 ± 1.72	0.025 ± 0.031
		48	32.6 ± 0.94	0.020 ± 0.021
Positive control (MMC)	2	24	33.4 ± 0.70	5.090 ± 0.513

PCE: polychromatic erythrocyte, NCE: normochromatic erythrocyte, SD: standard deviation, MNPCE: micronucleated polychromatic erythrocyte, MMC: mitomycin C. Significant difference from negative control.

**Table 3:** Linear range, regression equation, *r*^2^, LOD, and LOQ for the 12 markers determined by HPLC analysis (*n* = 3).

Analyte	Quantitative Wavelength (nm)	Linear Range (μg/mL)	Regression Equation	*r* ^2^	LOD (μg/mL)	LOQ (μg/mL)
**1**	280	0.31–20.00	*y* = 95,550.99*x* + 458.13	1.0000	0.03	0.09
**2**	280	0.31–20.00	*y* = 99,954.33*x* + 3602.82	1.0000	0.09	0.27
**3**	275	0.94–60.00	*y* = 15,130.59*x* + 2858.94	1.0000	0.11	0.32
**4**	275	0.31–20.00	*y* = 26,128.42*x* + 1517.82	1.0000	0.06	0.17
**5**	275	0.31–20.00	*y* = 65,883.81*x* + 3774.02	0.9999	0.06	0.18
**6**	275	0.78–50.00	*y* = 41,788.84*x* + 771.77	1.0000	0.11	0.34
**7**	275	0.31–20.00	*y* = 53,049.55*x* + 1924.39	1.0000	0.09	0.26
**8**	290	0.78–50.00	*y* = 156,240.95*x* + 26,984.77	0.9999	0.16	0.50
**9**	275	0.31–20.00	*y* = 64,648.61*x* − 2836.96	1.0000	0.07	0.20
**10**	255	0.31–20.00	*y* = 7375.30*x* − 645.92	0.9999	0.05	0.15
**11**	275	0.31–20.00	*y* = 93,965.82*x* + 4297.31	1.0000	0.06	0.17
**12**	220	0.31–20.00	*y* = 27,871.86*x* − 3292.83	0.9998	0.01	0.03

**Table 4:** Recovery test results (%) for the 12 markers determined using the HPLC method (*n* = 5).

Analyte	Spiked Conc. (μg/mL)	Measured Conc. (μg/mL)	Recovery (%)	SD	RSD (%)
**1**	1.00	0.97	96.99	0.76	0.78
	2.00	1.97	98.37	0.85	0.86
	4.00	3.82	95.39	0.33	0.35
**2**	1.00	0.99	99.08	0.64	0.65
	2.00	2.02	101.21	0.77	0.76
	4.00	4.02	100.53	0.39	0.39
**3**	4.00	4.03	100.84	2.37	2.35
	10.00	9.70	96.96	1.00	1.03
	20.00	19.74	98.71	1.15	1.16
**4**	1.00	0.98	98.44	1.34	1.36
	2.00	2.00	100.09	0.26	0.26
	4.00	4.05	101.28	2.15	2.12
**5**	1.00	1.01	100.99	1.39	1.38
	2.00	1.98	98.89	0.37	0.38
	4.00	4.03	100.85	1.55	1.53
**6**	4.00	4.04	101.04	0.88	0.87
	8.00	7.79	97.37	2.13	2.19
	16.00	15.91	99.44	0.15	0.15
**7**	1.00	0.97	96.71	1.51	1.56
	2.00	2.03	101.65	0.92	0.91
	4.00	4.17	104.31	0.35	0.34
**8**	2.00	2.04	102.05	1.28	1.25
	5.00	4.91	98.26	0.79	0.80
	10.00	10.34	103.44	0.55	0.53
**9**	1.00	1.00	100.13	2.58	2.57
	2.00	2.06	103.09	1.32	1.28
	4.00	4.07	101.68	0.47	0.46
**10**	1.00	1.02	102.22	1.56	1.53
	2.00	1.96	97.97	1.49	1.52
	4.00	4.13	103.33	1.28	1.24
**11**	1.00	1.02	101.91	0.22	0.22
	2.00	1.96	97.98	0.26	0.26
	4.00	3.83	95.74	0.16	0.17
**12**	1.00	1.00	99.73	1.60	1.61
	2.00	1.95	97.69	0.41	0.42
	4.00	4.02	100.52	0.29	0.29

**Table 5:** Precision data for the 12 markers determined using the HPLC method (*n* = 5).

Analyte	Conc. (μg/mL)	Intra-Day			Inter-Day		
		Measured Conc. (μg/mL)	Precision (RSD, %)	Accuracy (%)	Measured Conc. (μg/mL)	Precision (RSD, %)	Accuracy (%)
**1**	5.00	4.88	0.57	97.63	4.76	2.09	95.15
	10.00	10.14	0.36	101.43	10.21	0.63	102.07
	20.00	19.91	0.25	99.57	20.10	0.77	100.48
**2**	5.00	5.03	0.34	100.53	4.96	1.10	99.28
	10.00	10.06	0.15	100.58	9.95	0.94	99.53
	20.00	20.10	0.43	100.48	19.88	0.95	99.38
**3**	15.00	15.28	0.70	101.89	15.13	1.03	100.89
	30.00	30.45	0.26	101.49	30.21	0.89	100.69
	60.00	60.54	0.54	100.90	59.99	0.87	99.99
**4**	5.00	5.08	0.55	101.65	5.04	0.92	100.81
	10.00	10.15	0.20	101.47	10.06	0.88	100.63
	20.00	20.16	0.53	100.81	19.99	0.85	99.95
**5**	5.00	5.07	0.50	101.33	5.03	0.87	100.53
	10.00	10.11	0.20	101.10	10.03	0.70	100.31
	20.00	20.09	0.35	100.45	19.91	0.81	99.57
**6**	12.50	12.62	0.68	101.00	12.51	1.06	100.11
	25.00	25.37	0.21	101.47	25.14	0.86	100.58
	50.00	50.42	0.44	100.84	50.01	0.79	100.03
**7**	5.00	5.06	0.62	101.22	5.03	0.89	100.56
	10.00	10.13	0.29	101.32	10.05	0.83	100.48
	20.00	20.18	0.48	100.88	20.00	0.85	100.01
**8**	12.50	12.70	0.46	101.64	12.61	0.81	100.90
	25.00	25.39	0.21	101.56	25.21	0.67	100.82
	50.00	50.23	0.34	100.46	49.84	0.74	99.68
**9**	5.00	5.03	0.75	100.56	4.98	0.94	99.68
	10.00	10.11	0.39	101.06	9.99	0.99	99.89
	20.00	20.17	0.39	100.84	19.96	0.93	99.78
**10**	5.00	4.83	0.73	96.54	4.82	0.95	96.33
	10.00	9.99	0.41	99.91	9.96	0.44	99.62
	20.00	20.22	0.56	101.10	20.42	0.93	102.11
**11**	5.00	5.07	0.59	101.46	5.03	0.91	100.66
	10.00	10.14	0.28	101.45	10.06	0.80	100.65
	20.00	20.17	0.48	100.87	20.00	0.82	100.00
**12**	5.00	5.09	0.31	101.82	5.13	0.76	102.57
	10.00	10.28	0.09	102.82	10.30	0.35	102.99
	20.00	20.43	0.29	102.14	20.42	0.32	102.10

Accuracy = measured concentration/concentration × 100.

**Table 6:** Amounts of the 12 markers in the 70% methanol extracts from the SRT water decoctions determined by HPLC–PDA (*n* = 3).

Analyte	Amount (mg/g Freeze-Dried Sample)						Origin
	Batch 1		Batch 2		Batch 3		
	Mean ± SD (×10^–2^)	RSD (%)	Mean ± SD (×10^–2^)	RSD (%)	Mean ± SD (×10^–2^)	RSD (%)	
**1**	0.10 ± 0.06	0.59	0.10 ± 0.15	1.55	0.10 ± 0.04	0.43	PU
**2**	0.04 ± 0.02	0.55	0.04 ± 0.08	1.77	0.04 ± 0.08	1.78	PU
**3**	2.05 ± 1.71	0.83	2.07 ± 5.17	2.50	2.05 ± 2.16	1.06	GU
**4**	0.60 ± 0.74	1.24	0.61 ± 1.63	2.67	0.60 ± 0.17	0.28	GU
**5**	0.36 ± 0.12	0.34	0.36 ± 0.12	0.33	0.36 ± 0.13	0.35	CC
**6**	16.78 ± 7.01	0.42	16.86 ± 6.98	0.41	16.63 ± 10.92	0.66	SB
**7**	4.05 ± 1.61	0.40	4.08 ± 2.04	0.50	4.01 ± 2.61	0.65	SB
**8**	1.29 ± 0.19	0.15	1.29 ± 0.08	0.06	1.29 ± 0.21	0.16	CC
**9**	0.67 ± 0.07	0.10	0.68 ± 0.36	0.53	0.68 ± 0.22	0.32	SB
**10**	3.31 ± 3.77	1.14	3.36 ± 3.23	0.96	3.31 ± 3.51	1.06	GU
**11**	0.24 ± 0.07	0.28	0.24 ± 0.09	0.36	0.24 ± 0.06	0.24	SB
**12**	0.11 ± 0.14	1.35	0.11 ± 0.18	1.62	0.11 ± 0.13	1.26	AJ

PU, *P. umbellatus*; GU, *G. uralensis*; CC, *C. cassia*; SB, *S. baicalensis*; and AJ, *A. japonica*.

## Data Availability

All data are available in this article.

## Supplementary Materials

The following supporting information can be downloaded at: https://www.mdpi.com/article/10.3390/molecules27134066/s1. Figure S1: HPLC chromatogram obtained for the water extract from each raw herbal medicine and their major components. A, *B. falcatum*; B, *A. orientale*; C, *A. japonica*; D, *P. umbellatus*; E, *P. cocos*; F, *P. ternata*; G, *S. baicalensis*; H, *P. ginseng*; I, *G. uralensis*; J, *C. cassia*; and K, *Z. officinale*. Figure S2: Chemical structures of the 12 marker components in SRT. Table S1: Suitability of the system for simultaneous analysis of the 12 markers. Table S2: Repeatability of the retention times for the 12 markers determined using HPLC (*n* = 6). Table S3: Repeatability of the peak areas for the 12 markers determined using HPLC (*n* = 6). Table S4: Information about SRT and its composition. Table S5: HPLC analysis conditions for simultaneous determination of the 12 marker components in SRT.

## Sample Availability

Samples of the compounds are available from the authors.
