# Supplementary material for: In Vitro and In Vivo Genotoxicity Assessments and Phytochemical Analysis of the Traditional Herbal Prescription Siryung-Tang
Source: Molecules. 2022 Jun 24;27(13):4066. doi: 10.3390/molecules27134066 (PMC9268686; doi:10.3390/molecules27134066)
Supplement: Supplementary file 1 [file molecules-27-04066-s001.zip › molecules-1765751-supplementary.pdf]

Table S1. System suitability test of the 12 markers for simultaneous analysis

Marker	*k'*	*α*	*N*	*Rs*	T_f
**1**	2.00	1.81	55709	20.79	1.08
**2**	3.63	1.81	198609	20.79	1.11
**3**	5.58	1.03	471340	2.25	1.16
**4**	5.72	1.03	563040	2.25	1.12
**5**	8.05	1.03	492258	2.58	1.03
**6**	8.26	1.03	806873	2.58	1.24
**7**	9.59	1.07	969634	8.16	1.16
**8**	10.28	1.01	626786	1.57	1.05
**9**	10.41	1.01	1072555	1.57	1.11
**10**	12.01	1.04	1173115	5.64	1.19
**11**	12.46	1.04	1276118	5.64	1.04
**12**	13.66	1.10	1308608	14.26	1.01

k'; capacity factor, *α*; relative retention, *N*; theoretical plate number, *Rs*; resolution, and T_f; tailing factor 5-(Hydroxy-methyl)furfural (**1**), 3,4-dihydroxybenzaldehyde (**2**), liquiritin apioside (**3**), liquiritin (**4**), coumarin (**5**), baicalin (**6**), wogonoside (**7**), cinnamaldehyde (**8**), baicalein (**9**), glycyrrhizin (**10**), wogonin (**11**), and atractylenolide III (**12**).

Table S2. Repeatability for retention time of the 12 markers using HPLC (n = 6)

Analyte	No., Retention time (min)								
	1	2	3	4	5	6	Mean	SD	RSD (%)
**1**	8.40	8.39	8.40	8.39	8.39	8.39	8.39	0.01	0.07
**2**	12.94	12.93	12.94	12.93	12.92	12.93	12.93	0.01	0.06
**3**	18.39	18.38	18.39	18.38	18.38	18.38	18.38	0.01	0.04
**4**	18.79	18.77	18.79	18.77	18.77	18.77	18.77	0.01	0.05
**5**	25.28	25.26	25.28	25.26	25.26	25.26	25.26	0.01	0.04
**6**	25.89	25.87	25.89	25.87	25.87	25.87	25.87	0.01	0.04
**7**	29.58	29.57	29.58	29.56	29.56	29.56	29.57	0.01	0.04
**8**	31.49	31.48	31.49	31.47	31.46	31.47	31.47	0.01	0.04
**9**	31.87	31.86	31.87	31.85	31.85	31.85	31.86	0.01	0.04
**10**	36.26	36.25	36.26	36.24	36.24	36.24	36.25	0.01	0.03
**11**	37.59	37.58	37.59	37.57	37.57	37.57	37.58	0.01	0.03
**12**	40.92	40.90	40.91	40.89	40.89	40.89	40.90	0.01	0.03

5-(Hydroxy-methyl)furfural (**1**), 3,4-dihydroxybenzaldehyde (**2**), liquiritin apioside (**3**), liquiritin (**4**), coumarin (**5**), baicalin (**6**), wogonoside (**7**), cinnamaldehyde (**8**), baicalein (**9**), glycyrrhizin (**10**), wogonin (**11**), and atractylenolide III (**12**).

Table S3. Repeatability for peak area of the 12 markers using HPLC (n = 6)

Analyte	No., Peak area								
	**1**	**2**	**3**	**4**	**5**	**6**	**Mean**	**SD**	**RSD (%)**
**1**	2181315	2190273	2167605	2168303	2194796	2213853	2186024.17	17577.96	0.80
**2**	2129581	2132364	2116548	2117639	2144098	2159151	2133230.17	16281.56	0.76
**3**	574674	577107	572111	572237	580176	585492	576966.17	5180.82	0.90
**4**	985415	987930	980831	982474	995695	1002893	989206.33	8495.90	0.86
**5**	2535502	2542157	2523794	2521075	2553139	2574012	2541613.17	19796.52	0.78
**6**	1678063	1681535	1669374	1669779	1690902	1705322	1682495.83	13756.24	0.82
**7**	2183180	2188653	2172448	2173183	2200900	2220859	2189870.50	18494.31	0.84
**8**	5797198	5813825	5772857	5765014	5835708	5883737	5811389.83	43973.45	0.76
**9**	2473468	2480344	2462550	2465143	2496440	2513273	2481869.67	19618.41	0.79
**10**	1111409	1112251	1106050	1105236	1120720	1131211	1114479.50	9897.27	0.89
**11**	2064345	2068620	2054378	2052897	2055607	2073942	2061631.50	8636.55	0.42
**12**	2212860	2212805	2206683	2203328	2227744	2244486	2217984.33	15447.99	0.70

5-(Hydroxy-methyl)furfural (**1**), 3,4-dihydroxybenzaldehyde (**2**), liquiritin apioside (**3**), liquiritin (**4**), coumarin (**5**), baicalin (**6**), wogonoside (**7**), cinnamaldehyde (**8**), baicalein (**9**), glycyrrhizin (**10**), wogonin (**11**), and atractylenolide III (**12**).

Table S4. Information and composition of SRT

Herbal medicine	Scientific name	English name	Family	Used part	Origin	Amount (g)
Bupleuri Radix	*Bupleurum falcatum* L.	Bupleurum Root	Apiaceae	Root	Cheongsong, Korea	767.0
Alismatis Rhizoma	*Alisma orientale* Juzep.	Alisma Rhizome	Alismataceae	Tuber	Imsil, Korea	622.6
Atractylodis Rhizoma Alba	*Atractylodes japonica* Koidz.	Atractylodes Rhizome White	Compositae	Rhizome	Uljin, Korea	359.2
Polyporus	*Polyporus umbellatus* Fires	Polyporus Sclerotium	Polyporaceae	Sclerotium	China	359.2
Poria Sclerotium	*Poria cocos* Wolf	Poria	Polyporaceae	Sclerotium	Bonghwa, Korea	359.2
Pinelliae Tuber	*Pinellia ternata* (Thunb.) Makino	Pinellia Tuber	Araceae	Tuber	China	335.0
Scutellariae Radix	*Scutellaria baicalensis* Georgi	Scutellaria Root	Lamiaceae	Root	Yeosu, Korea	287.6
Ginseng Radix	*Panax ginseng* C.A.Mey.	Ginseng	Araliaceae	Root	Punggi, Korea	287.6
Glycyrrhizae Radix et Rhizoma	*Glycyrrhiza uralensis* Fisch.	Licorice	Leguminosae	Root and rhizome	China	287.6
Cinnamomi Cortex	*Cinnamomum cassia* (L.) J.Presl	Cinnamom Bark	Lauraceae	Bark	Veitanam	143.2
Zingiberis Rhizoma Recens	*Zingiber officinale* Rosc.	Raw Ginger	Zingiberaceae	Rhizome	Seosan, Korea	191.8
					Total (g)	4000.0

Table S5. HPLC analysis conditions for simultaneous determination of the 12 marker components in SRT

**HPLC analysis parameter**			
HPLC system	Prominence LC-20A series (Shimadzu, Kyoto, Japan)		
Detector	Photo-diode array detector		
Detection wavelength (nm)	220, 255, 275, 280, and 290		
Column	SunFire C18 (4.6 × 250 mm, 5 µm, Waters, Milford, MA, USA)		
Column oven temperature (°C)	40.0		
Flow rate (mL/min)	1.0		
Injection volume (µL)	10.0		
Mobile phase	A: 0.1% (v/v) formic acid in distilled water<br>B: 0.1% (v/v) formic acid in acetonitrile		
Gradient elution	Time (min)	A (%)	B (%)
	0	95	5
	40	40	60
	50	0	100
	55	0	100
	60	95	5
	70	95	5

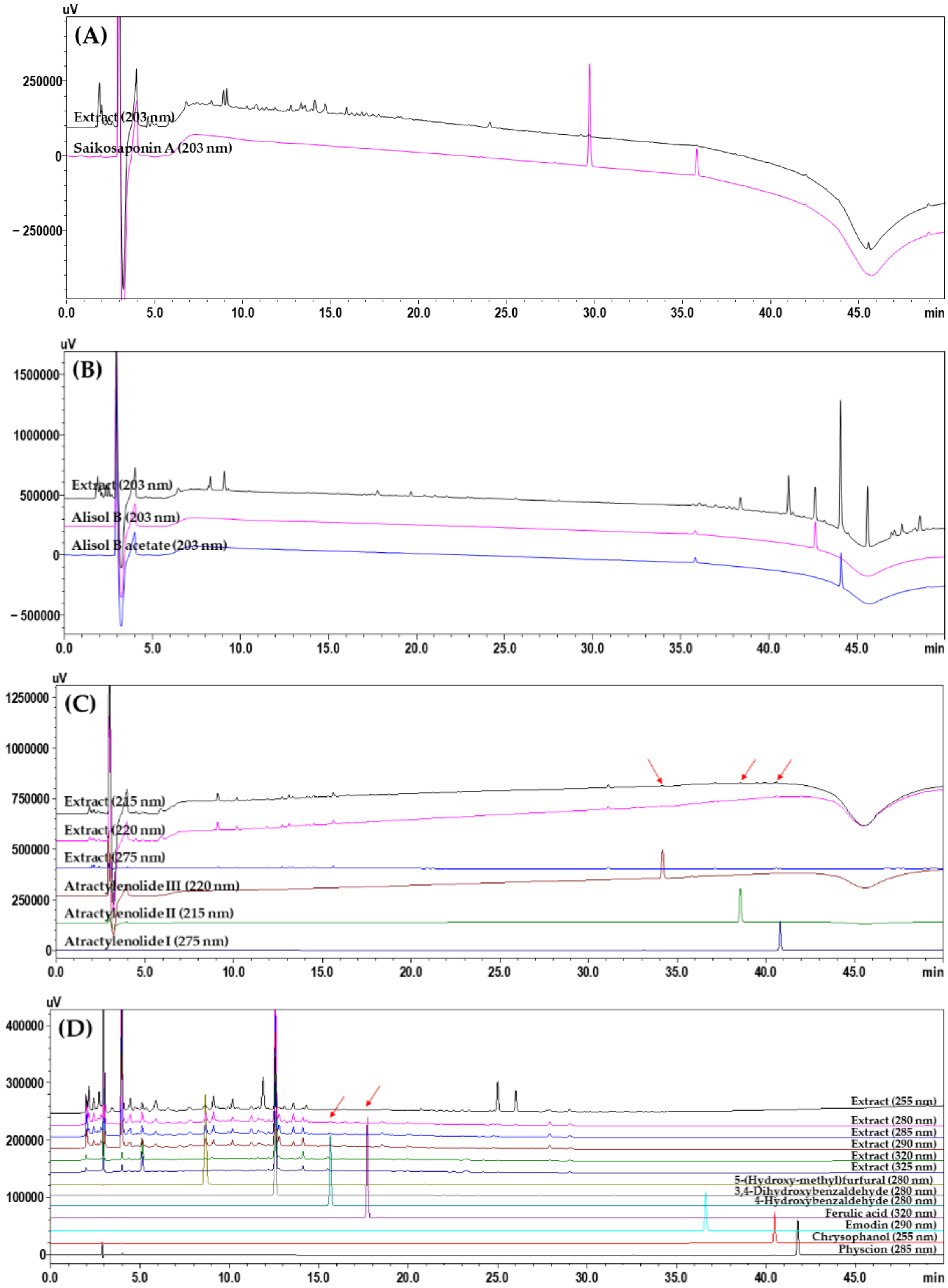
(A)
uV
Extract (203 nm)
Saikosaponin A (203 nm)
min
(B)
Extract (203 nm)
Alisol B (203 nm)
Alisol B acetate (203 nm)
(C)
Extract (215 nm)
Extract (220 nm)
Extract (275 nm)
Atractylenolide III (220 nm)
Atractylenolide II (215 nm)
Atractylenolide I (275 nm)
(D)
Extract (255 nm)
Extract (280 nm)
Extract (285 nm)
Extract (290 nm)
Extract (320 nm)
Extract (325 nm)
5-(Hydroxy-methyl)furfural (280 nm)
3,4-Dihydroxybenzaldehyde (280 nm)
4-Hydroxybenzaldehyde (280 nm)
Ferulic acid (320 nm)
Emodin (290 nm)
Chrysophanol (255 nm)
Physcion (285 nm)

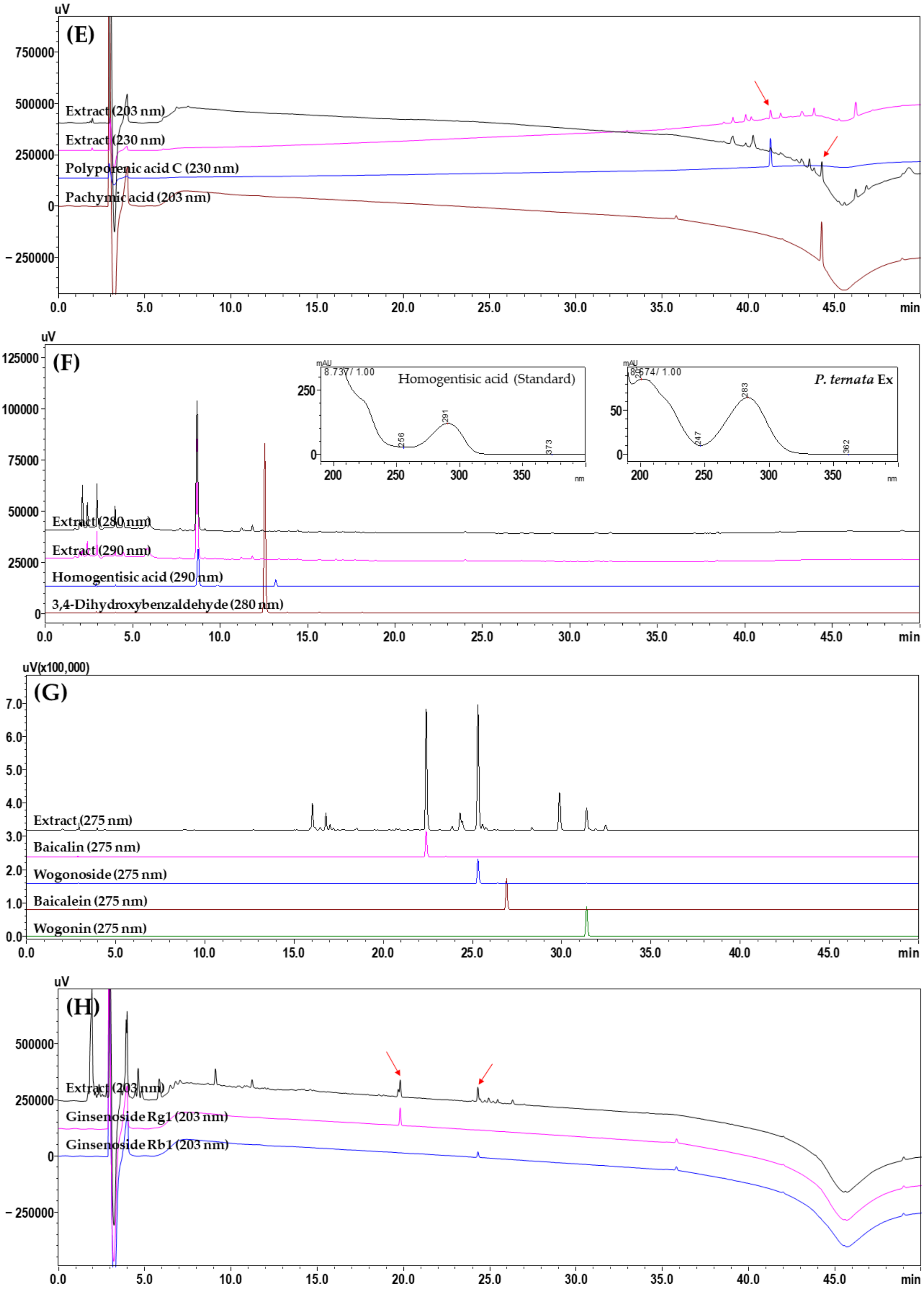
(E)
Extract (203 nm)
Extract (230 nm)
Polyporenic acid C (230 nm)
Pachymic acid (203 nm)
uV
min
(F)
Extract (280 nm)
Extract (290 nm)
Homogentisic acid (290 nm)
3,4-Dihydroxybenzaldehyde (280 nm)
Homogentisic acid (Standard)
P. ternata Ex
uV
min
(G)
Extract (275 nm)
Baicalin (275 nm)
Wogonoside (275 nm)
Baicalein (275 nm)
Wogonin (275 nm)
uV(x100,000)
min
(H)
Extract (203 nm)
Ginsenoside Rg1 (203 nm)
Ginsenoside Rb1 (203 nm)
uV
min

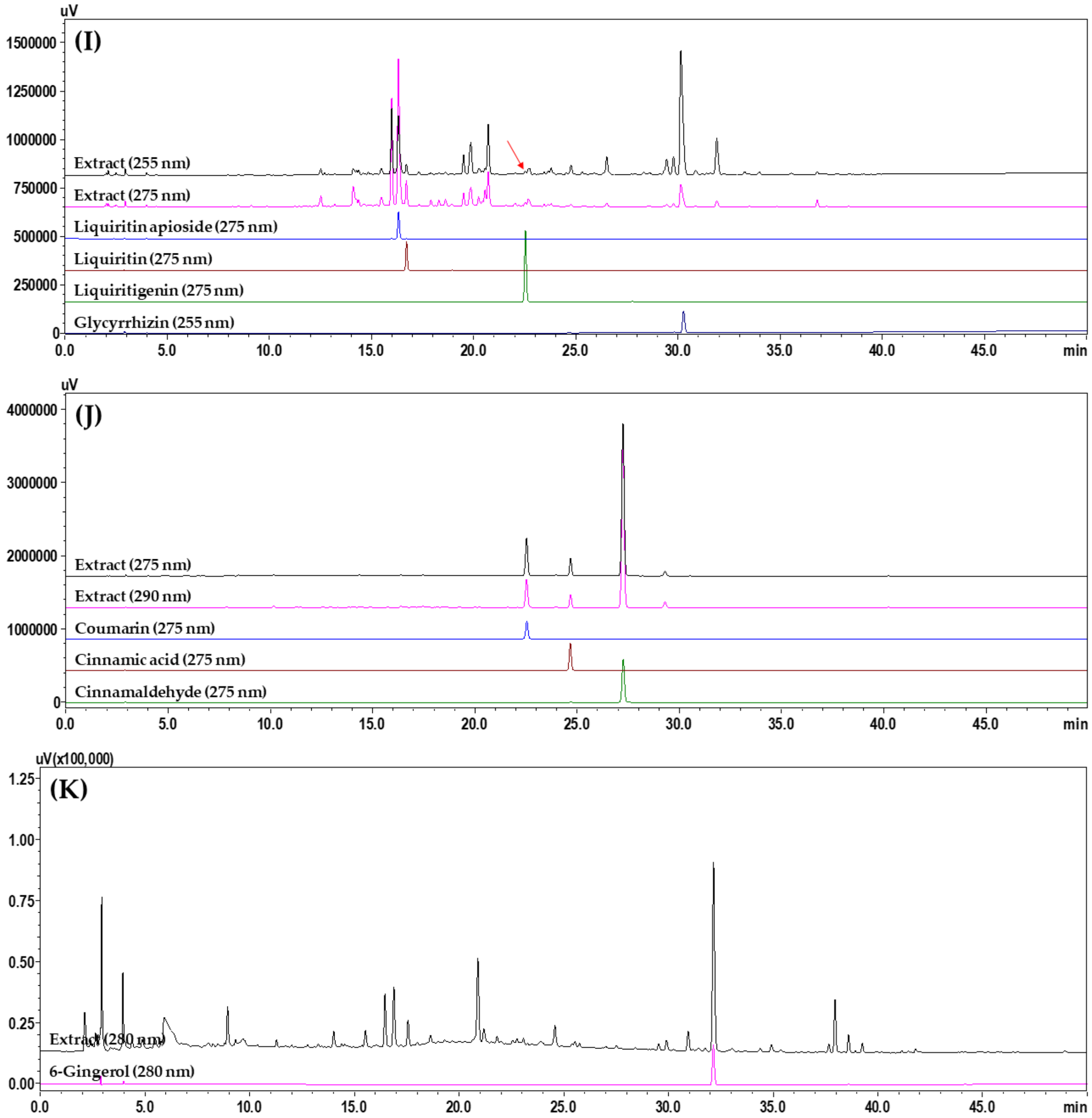

Figure S1. HPLC chromatogram of water extract of each raw herbal medicine and their major components. A, *B. falcatum*; B, *A. orientale*; C, *A. japonica*; D, *P. umbellatus*; E, *P. cocos*; F, *P. ternata*; G, *S. baicalensis*; H, *P. ginseng*; I, *G. uralensis*; J, *C. cassia*; and K, *Z. officinale*.

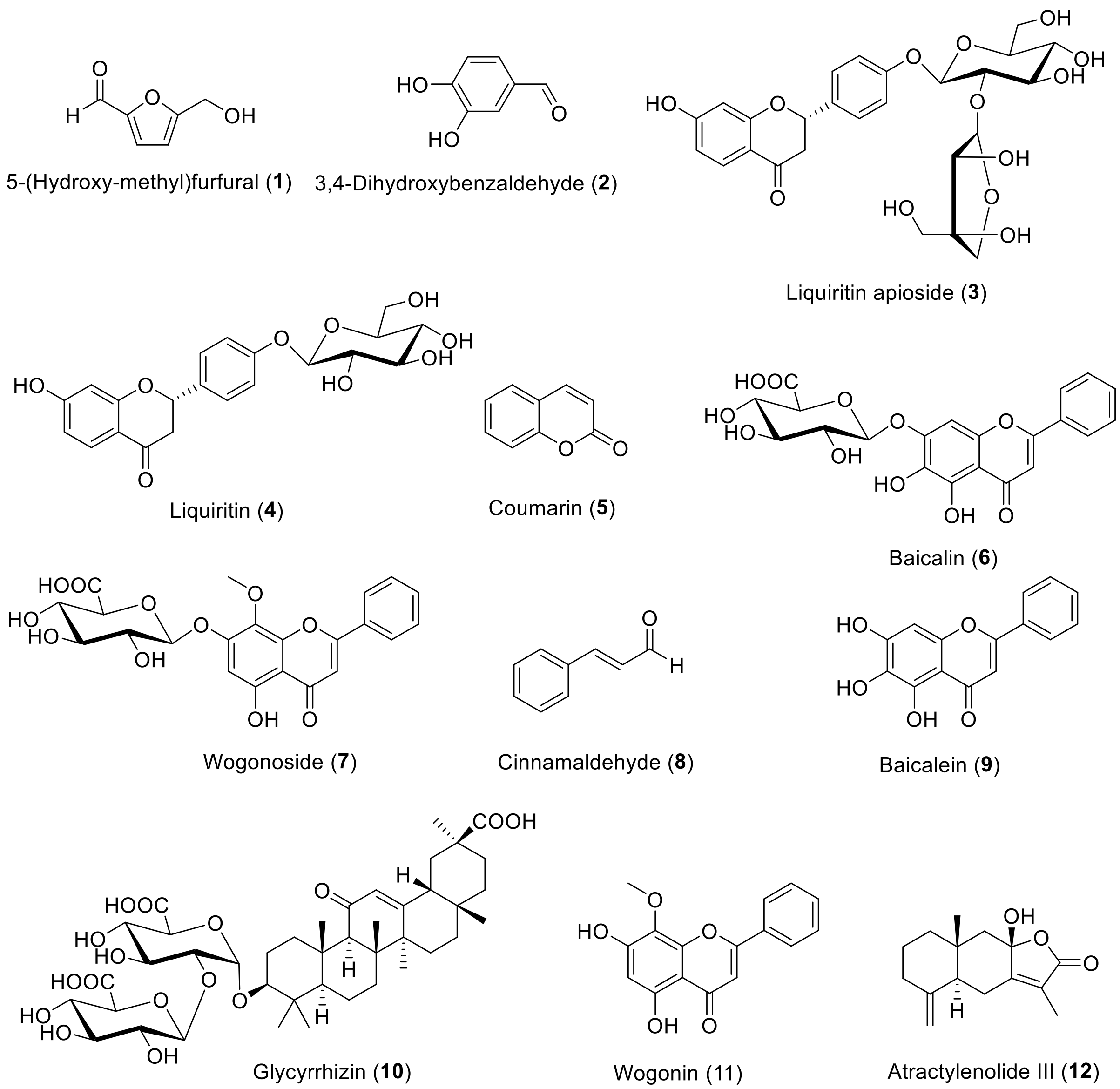

Figure S2. Chemical structures of the 12 marker components in SRT.
